# Exploring the Early Endometrial–Blastocyst Interactome in Endometriosis: An Integrative Study

**DOI:** 10.3390/biomedicines13112588

**Published:** 2025-10-23

**Authors:** Ana Schafir, Lourdes Materazzi, Lara Castagnola, Agostina Occhiuzzi, Daniel Paparini, Lautaro Tessari, Lautaro Fierro, Marcela Irigoyen, Antonio Cattaneo, Diego Gnocchi, Soledad Gori, Esteban Grasso, Rosanna Ramhorst

**Affiliations:** 1 Consejo Nacional de Investigaciones Científicas y Técnicas, Laboratorio de Inmunofarmacología, Instituto de Química Biológica de la Facultad de Ciencias Exactas y Naturales (IQUIBICEN), Universidad de Buenos Aires, Buenos Aires C1428EGA, Argentina; ana_schafir@outlook.com (A.S.); msoledadgori@qb.fcen.uba.ar (S.G.); 2 Fertilis Medicina Reproductiva, Buenos Aires B1609JEC, Argentinalautaro.tessari@fertilis.com.ar (L.T.); lautaro.fierro@fertilis.com.ar (L.F.); marcela.irigoyen@fertilis.com.ar (M.I.); diego.gcnocci@fertilis.com.ar (D.G.)

**Keywords:** endometriosis, implantation, interactome, inflammation, infertility

## Abstract

**Background:** Background: Endometriosis affects 10% of women of reproductive age. Despite the well-known association between endometriosis and infertility, the mechanisms underlying this association remain to be elucidated. **Methods:** Implantation and pregnancy success rates were evaluated by a retrospective study of patients that underwent IVF using euploid embryos comparing healthy vs. endometriosis patients. To study the early embryo–endometrial dialogue, an interactome network was constructed using public RNAseq data from normal secretory-phase endometrial samples and day-5 blastocyst. Public bulk and single-cell RNAseq data from endometrial samples of endometriosis patients were used to detect alterations in the interactome. **Results:** Endometriosis patients required significantly more IVF attempts compared to those without endometrial pathologies; however, once pregnancy was achieved, the evolution of both groups was similar. The interactome network between normal endometrium and day-5 blastocyst showed a significant enrichment of pathways associated with tissue remodelling, angiogenesis, and immune regulation, which were altered in endometriosis patients. Endometriosis patients also presented an increased frequency and activation of NK, CD4^+^, and CD8^+^ cells, which interfere with embryo–endometrial dialogue. **Conclusions:** We identified key molecular processes affected by endometriosis specifically involved in the early interaction between the blastocyst, decidual, and resident immune cells, that may underline the reported fertility problems associated with endometriosis.

## 1. Introduction

Endometriosis is a chronic, debilitating, and estrogen-dependent inflammatory gynecological disorder characterized by the presence and growth of endometrial-like tissue outside the confines of the uterine cavity. Affecting approximately 10% of women of reproductive age worldwide, which accounts for around 190 million individuals, endometriosis presents a wide range of distressing symptoms [1,2] and systemic inflammatory effects, associated with pelvic pain and infertility [3,4].

In fact, infertility is a significant challenge for 30–50% of affected women, and mechanisms underlining endometriosis-associated infertility are still being debated. In this sense, plenty of evidence indicates that endometrial pathogenesis could be generated by an exacerbated inflammation in the uterus and ovaries, impacting endometrial receptivity and ovarian reserve, leading to infertility [5]. It has been reported that endometriosis is linked with an aberrant gene expression in the endometrium associated with an increased production of inflammatory cytokines and chemokines, resulting in a differential recruitment and differentiation of immune cells, which reshapes immune response in the uterus and ovarian microenvironment [6]. Furthermore, the decidualization program is altered due to estradiol causing an increase in prostaglandin E2 production and resistance to progesterone, impacting the implantation rate [7].

Since decidualized cells display an active role during embryo implantation associated with an initial sterile and physiological inflammatory response [8,9,10]; the exacerbation of the inflammatory response during the peri implantation period might negatively impact the implantation rate. In this sense, an exacerbated inflammatory response during the peri-implantation period can compromise endometrial receptivity and fertility in women [11,12].

However, the receptive endometrium represents just one of the interlocutors, the other one in this dialogue is the embryo, which produces soluble ligands that will interact with receptors expressed in endometrial cells targeting different autocrine and paracrine pathways [13,14,15]. Providing new clues to understand the molecular basis of the embryo–endometrial dialogue, we evidenced that soluble factors secreted by high quality human blastocysts promote implantation [16,17].

Currently, most data are focused on the maternal immune response in the endometrium from patients with endometriosis, but the primary role of the blastocyst-produced mediators in shaping this immune microenvironment is an open question. Even more, how the embryo–endometrium interaction is modulated in patients with endometriosis still needs to be thoroughly studied.

This leads to the following questions: How does endometriosis alter the embryo–endometrium interaction? Which are the main pathways affected that will negatively impact embryo implantation? Which are the main immune cells differentially modulated during the dialogue between the endometrium of an endometriosis patient and the blastocyst?

Here, we investigate the role of the embryo–endometrial dialogue in shaping the receptive immunomodulatory milieu and how these interactions are altered in patients with endometriosis. We evaluate the implantation rates in patients with endometriosis after ART (Assisted Reproductive Technology) procedures and we focus on the production of soluble factors derived from euploid-blastocysts and their interaction with membrane proteins in endometrial cells from patients with endometriosis.

## 2. Materials and Methods

### 2.1. Patient’s Clinical Data

Initial clinical information was obtained as a retrospective observational study using anonymized records of patients from 2019 to 2023 that underwent in vitro fertilization treatments (IVF) and used euploid embryos. For inclusion, patients must be between 25 and 50 years old and complete at least one IVF/ICSI treatment using only euploid embryos. Patients with other endometrial alterations besides endometriosis, such as adenomyosis, polyps, and myomas, or with ovarian alterations were excluded. All endometriotic patients were surgically treated for endometriosis prior to the embryo transfer. Embryo ploidy was assessed through preimplantation genetic testing for aneuploidy (PGT-A). Briefly after controlled ovarian stimulation and ultrasound-guided follicular aspiration, oocytes were fertilized via IVF or ICSI and cultured until the blastocyst stage (day 5–6). Trophectoderm biopsy was performed using laser-assisted zona pellucida opening. Samples were then sent to a local genetic diagnostic laboratory where DNA amplification was performed using the Yikon Genomics kit (Yikon Genomics Ltd, Shanghai, China), followed by next-generation sequencing (NGS) on an Illumina MiSeq^®^ platform (Illumina Inc, San Diego, CA, USA). Bioinformatic analysis enabled the detection of chromosomal gains and losses, classifying embryos as euploid or aneuploid.

Patients were classified as healthy patients (Control, *n* = 63) or endometriotic patients (*n* = 31). No significant difference was observed on age (control 39.7 ± 5.1; endometriosis 38.9 ± 4.7), endometrial thickness (control 9.36 ± 1.35; endometriosis 9.28 ± 1.21), embryo transfer day, or endometrial preparation. Statistical comparison between these two groups was performed using Mann–Whitney U test.

### 2.2. Data Source for Bioinformatics Analysis

Publicly available transcriptomic datasets were retrieved from the Gene Expression Omnibus (GEO). Bulk RNA-sequencing data included GSE141549 [18], comprising 12 secretory-phase eutopic endometrial samples from patients with severe endometriosis and 12 independent control samples from healthy individuals in the same menstrual phase, and GSE18290 [19] which contained three human embryo samples at day 5 of development. Single-cell RNA-sequencing data were obtained from GSE213216 [20], with 4 secretory-phase endometrial samples from healthy controls, and GSE214411 [21], with secretory-phase eutopic endometrial samples from 10 patients with severe endometriosis and 3 with minimal or mild endometriosis.

### 2.3. Bulk RNA-Seq Data Analysis

Gene expression data from datasets GSE141549 and GSE18290 were processed to identify expressed genes. A gene was considered expressed if it met two criteria simultaneously: (1) its median expression across all samples in the dataset exceeded the global median expression value of that dataset, and (2) it showed non-zero expression in more than 50% of the samples. This dual threshold was applied to ensure both relative abundance and consistent detectability across samples.

Expressed genes were further filtered based on the subcellular localization of their protein products. For endometrial samples, we retained only genes annotated as encoding plasma membrane receptors; for embryo samples, we retained only genes annotated as encoding secreted proteins. Protein localization was annotated using manually curated entries from the UniProtKB/Swiss-Prot database, specifically those associated with the controlled vocabulary terms “plasma membrane” and “secreted” under the cellular component annotation. This filtering strategy was designed to capture potential ligand–receptor interactions relevant to embryo–endometrium communication.

Differential expression analysis was performed on endometrial samples using the Comparative Markers Selection module from GenePattern platform (version 3.9) [22]. Genes were considered differentially expressed if they met the thresholds of fold change > 2 and false discovery rate FDR < 0.01.

### 2.4. Single-Cell (Sc)RNAseq Data Analysis

Single-cell RNAseq data was imported into R using the library Seurat. Each dataset was individually normalized and variance-stabilized using the sctransform method, which performs regularized negative binomial regression to account for technical noise and sequencing depth. Then, the selected samples were integrated using Seurat [23]. Cell types were identified using SingleR referencing the Human Primary Cell Atlas (HPCA). Cells classified as immune were further annotated using the Database of Immune Cell Expression (DICE), allowing for refined identification of B cells, monocytes/macrophages, NK cells, CD4^+^ T cells, and CD8^+^ T cells [24]. For each sample, the number of each immune cell type was counted and the proportion was calculated as a proportion relative to the total number of cells. To assess gene expression within each immune cell population, the average expression of each gene was calculated across all cells of the same type within each sample. These gene sets were then analyzed using the same pipeline described for bulk RNA-seq data.

### 2.5. Construction of Protein–Protein Interaction Networks

Protein–protein interaction networks were constructed to model potential communication between endometrial membrane receptors and soluble ligands expressed by the embryo or by resident immune cell populations. Networks were generated using the STRING database, configured to display physical interactions only—defined as experimentally supported or predicted direct protein–protein contacts, since these are more likely to represent biologically relevant ligand–receptor interactions in vivo. We applied a minimum confidence score of 0.7 (high confidence), as recommended by STRING 12.0 documentation to reduce false positives while retaining sufficient coverage of known interactions [25].

Following network generation, manual curation was performed to validate ligand–receptor pairs by reviewing the available literature for experimental evidence of receptor–ligand interactions. Although all predicted interactions in the STRING network met high-confidence criteria and were supported by experimental data, many involved proteins located within the same cellular component (e.g., both extracellular or both membrane-bound), which may not reflect the ligand–receptor communication we aim to study here. Therefore, we retained only those interactions for which published evidence explicitly described a receptor–ligand relationship between the two proteins. During the curation we were able to find reported evidence at protein level expression at endometrial tissue for 115 of the 126 endometrial receptors.

Pathway enrichment analysis of the resulting networks was conducted within the STRING 12.0 platform, using Reactome (release 93), KEGG, and Gene Ontology Biological Process annotations to identify overrepresented biological processes [26,27].

### 2.6. Enrichment Analysis

Gene Set Enrichment Analysis (GSEA) was performed to identify biological pathways differentially represented in endometrial samples from patients with severe endometriosis [28]. The analysis was applied to both bulk RNA-sequencing data and immune cell populations identified from single-cell RNA-sequencing datasets. Gene sets were derived from the Reactome (r.93) and Gene Ontology Biological Process (GOBP) databases. Pathways were considered significantly enriched if they met the thresholds of FDR < 0.25 and normalized enrichment score |NES| > 1.5. These thresholds were selected based on GSEA guidelines for exploratory biological discovery, allowing for the detection of moderate but biologically meaningful enrichment signals while controlling for false positives.

For bulk RNA-seq data, enriched pathways were manually grouped into broader biological categories based on functional similarity, enabling the identification of higher-order processes such as inflammation, stress response, and cell death. This grouping was performed through biological criteria and literature-based associations including hierarchy structure of pathway annotations. Specifically, pathways annotated for cytokine signalling, leukocyte activation, and chemotaxis were grouped under “inflammation”; pathways involving unfolded protein response, oxidative stress, and hypoxia were grouped under “stress response”; and pathways regulating apoptosis, necroptosis, and autophagy were grouped under “cell death”. No automated clustering tools were used as the classification relied on semantic and functional interpretation of pathway annotations.

## 3. Results

### 3.1. Endometriosis Effects on Implantation and Pregnancy Outcome

**Figure 1:**
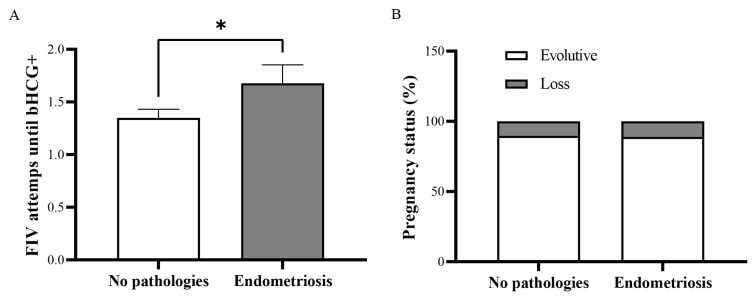
Patients with endometriosis require more IVF attempts until implantation (bHCG+) is achieved. (**A**) Mean ± SEM of IVF attempts until bHCG+ results in endometriosis patients (*n* = 31) and control (*n* = 63). (**B**) Evolution of pregnancy within bHCG+ patients with endometriosis (*n* = 27) and control (*n* = 63). * *p* < 0.05, Mann–Whitney U test.

To start evaluating the effects of endometriosis in fertility, we performed a retrospective study on patients that underwent IVF with PGT-studied (euploid) blastocysts in order to only evaluate the endometrial factor, comparing patients with no endometrial alterations, such as adenomyosis, polyps, and myomas, (control) with patients treated for endometriosis. We found that patients that had endometriosis required significantly more attempts to achieve implantation (bHCG+) than patients with no endometrial alterations (Figure 1A). However, once implantation was achieved, there was no significant difference in the pregnancy outcome (Figure 1B). This suggests that the negative effect of endometriosis in fertility might involve the early stages of implantation.

### 3.2. Endometrium–Embryo Interactome

**Figure 2:**
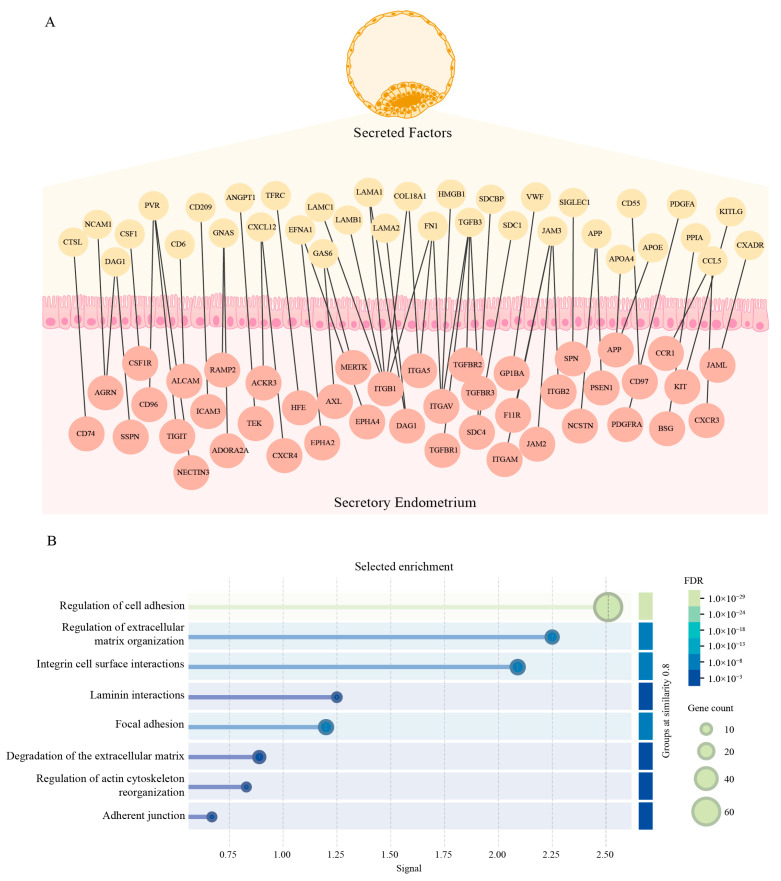
Endometrium–embryo interactions are enriched in genes involved in cell-to-cell adhesion and tissue remodelling. (**A**) Protein–protein interaction graph of membrane receptors expressed in endometrial biopsies during the secretory phase (GSE141549) and soluble factors expressed in day 5 embryos (GSE18290). Gene expression was assessed at the transcriptomic level (mRNA), and interactions were inferred at the protein level using STRING. Nodes represent proteins annotated for cell adhesion and tissue remodelling and edges denote experimentally supported interactions. (**B**) Functional enrichment of the interaction network in pathways (Reactome and KEGG) associated with cell adhesion and tissue remodelling (Strength > 0.7 and FDR < 0.001).

**Figure 3:**
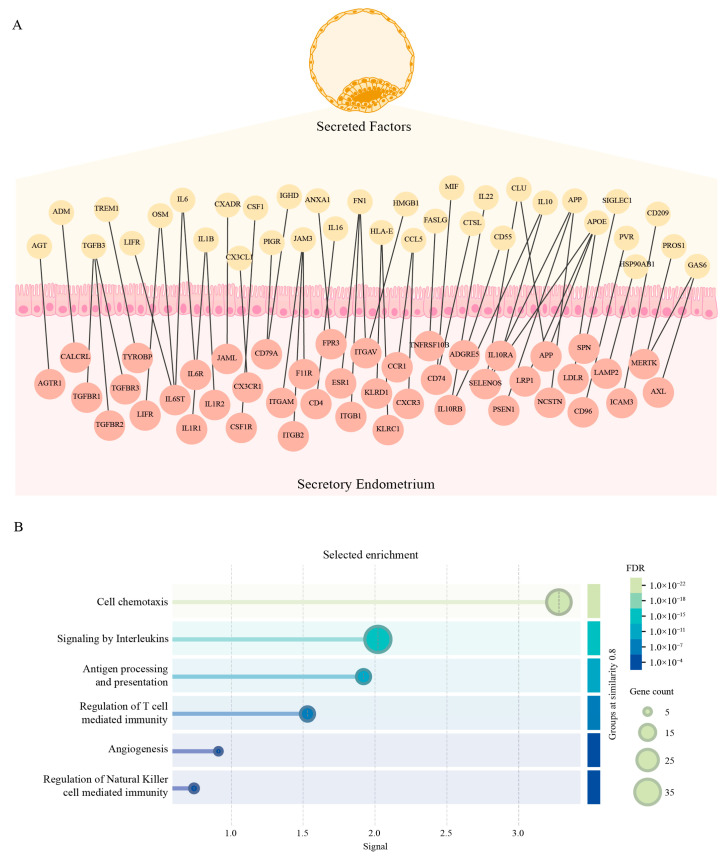
Endometrium–embryo interactions are enriched in genes involved in endometrial inflammatory response. (**A**) Protein–protein interaction graph of membrane receptors expressed in endometrial biopsies during the secretory phase (GSE141549) and soluble factors expressed in day 5 embryos (GSE18290). Gene expression was assessed at the transcriptomic level (mRNA), and interactions were inferred at the protein level using STRING. Nodes represent proteins annotated for inflammation and edges denote experimentally supported interactions. (**B**) Enrichment of the interaction network in pathways (Reactome and KEGG) associated with inflammation (Strength > 0.7 and FDR < 0.001).

In order to study the early interaction between the blastocyst and the endometrium, we opted for a bioinformatics approach to generate an interactome network. For the blastocyst’s side, we used transcriptomic data from day-5 embryo samples from GSE18290, selecting only genes that were secreted according to Gene Ontology. For the endometrial side, we used transcriptomic data from endometrial biopsies of healthy donors on the secretory phase from GSE141549, selecting only membrane receptors. On both cases, we only considered genes that were expressed in at least half of the samples and that their median expression was greater than the general median to take a conservative approach. A total of 857 genes were selected for the embryo’s side and 456 genes for the endometrial side and interactions were inferred at the protein level using STRING, only considering experimental supported interactions (Figure S1 and Table S1). Interestingly, when we analyzed pathways that were enriched in the network, we found several genes involved in cell adhesion and tissue remodelling (Figure 2A) as well as in regulation of the immune response (Figure 3A). Furthermore, several specific pathways involved in these processes were also significantly enriched (Figure 2B and Figure 3B). These results suggest that during the early stages of implantation, the blastocyst secretes factors that the endometrium detects through its receptors, highlighting the role of the dialogue between the blastocyst and the endometrium in the modulation of these three key processes. A detailed list of the enriched pathways and the corresponding genes involved is provided in Table S2.

Another interesting finding is that we detected factors that the blastocyst may secrete that could exert regulatory effects on the endometrium through indirect or receptor-independent mechanisms (Figure S2). These decoy factors include soluble isoforms of cytokine receptors and other regulatory proteins that do not require membrane-bound counterparts to exert their function. Instead, they bind to soluble ligands in the extracellular space, modulating their availability and activity, and thereby influencing the endometrial microenvironment. As these interactions do not involve membrane-bound receptor–ligand pairs, they are not represented in the interactome network, which was constructed based on physical protein–protein interactions between membrane receptors and secreted ligands. Nevertheless, these genes were retained for pathway enrichment analysis, as they are functionally involved in immune regulation and tissue remodelling processes relevant to implantation.

### 3.3. Interference of Endometriosis in the Endometrial–Embryo Dialogue

**Figure 4:**
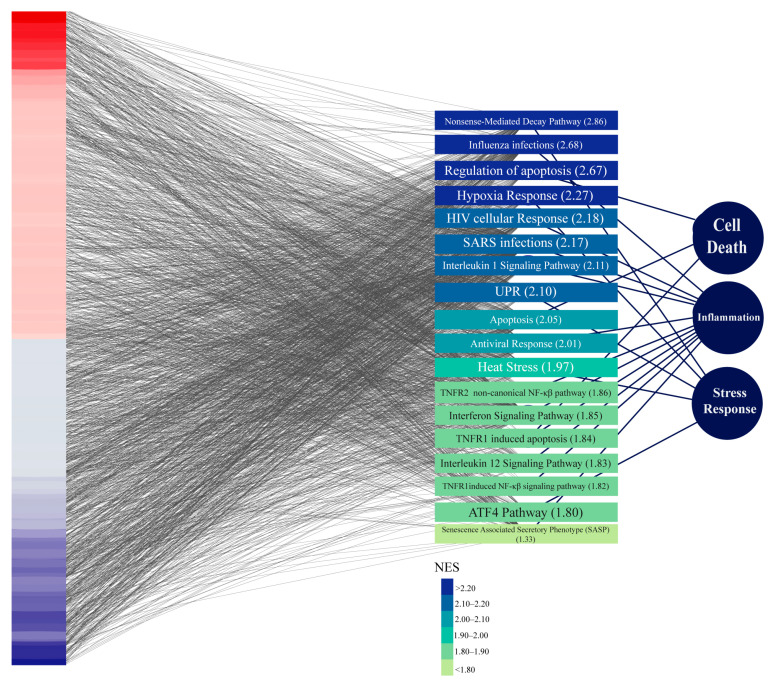
Transcriptomic profiling of eutopic endometrial tissue from patients with severe endometriosis revealing functional enrichment in inflammation, stress response, and cell death pathways. Bulk RNA-sequencing data from secretory-phase endometrial biopsies from patients with severe endometriosis and healthy controls were analyzed for differential gene expression. (**Left**) Heatmap visualizing the fold change in gene expression between groups, where red is higher expression in endometriosis while blue in healthy controls. (**Middle**) Reactome pathways enriched among differentially expressed genes in endometriosis. (**Right**) Higher-order biological processes grouping the enriched pathways.

To further investigate our initial clinical observation, we then evaluated which endometrial genes were modulated in the interactome network in patients with endometriosis. Considering that in our cohort none of the patients presented endometriosis lesions within the endometrium, we compared the expression of the control group with samples from eutopic endometrium on the secretory phase from patients with severe endometriosis from GSE141549. We found 3831 genes upregulated and 3420 genes downregulated in the endometrium from endometriosis patients. Furthermore, using GSEA, we identified that the most enriched pathways are involved in cell death, inflammation, and stress response (Figure 4), indicating that the endometrium of endometriosis patients is altered even if the lesion is not present.

**Figure 5:**
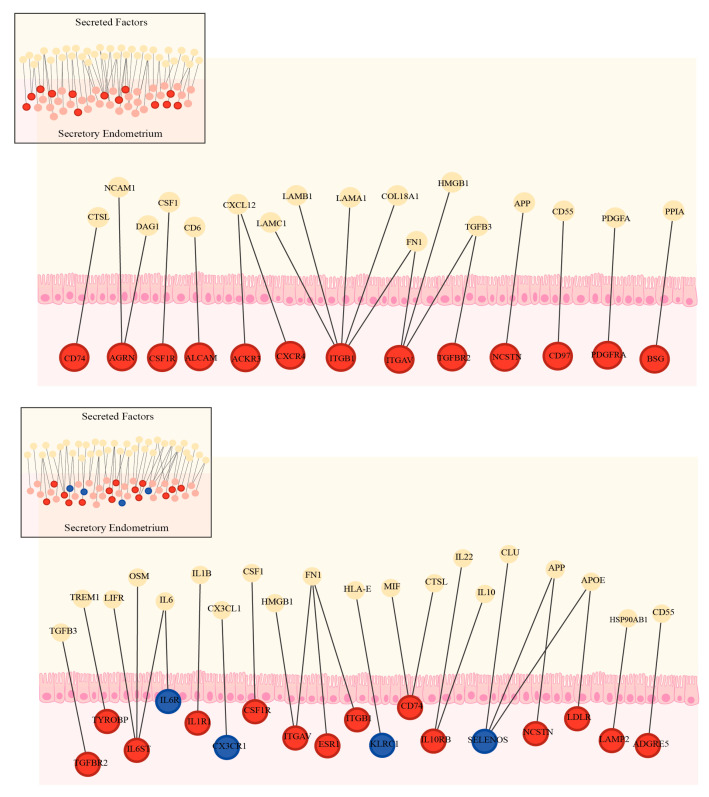
Differential gene expression in the eutopic endometrium of patients with severe endometriosis intersects with endometrium–embryo interaction networks. Transcriptomic analysis of secretory-phase endometrial biopsies identified up-regulated (red) and down-regulated (blue) genes in patients with severe endometriosis (|FC| > 2 and FDR < 0.01). These genes were mapped onto the endometrium–embryo interaction networks. (**Top**) Network interactions associated with cell adhesion and tissue remodelling. (**Bottom**) Network interactions associated with inflammation (FDR < 0.01).

When we evaluated modulations of the endometrial genes on the interactome, we found that 13 out of 43 genes (30.2%, Figure 5 top) involved in tissue remodelling and cell adhesion, and 18 out of 44 genes (40.9%, Figure 5 bottom) involved in immune response were significantly modulated, suggesting that the dialogue between the blastocyst and the endometrium could be severely affected in patients with endometriosis. The effect of endometriosis on the complete interactome is shown in Figure S3.

### 3.4. Role of the Immune Cells in the Effects of Endometriosis on the Interactome

**Figure 6:**
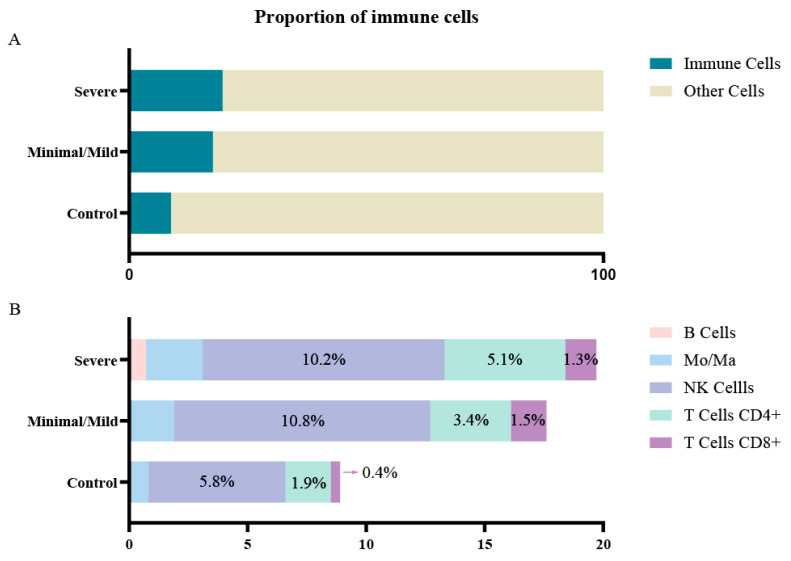
Single-cell transcriptomic analysis reveals altered immune cell proportions in the eutopic endometrium of patients with severe endometriosis. Single-cell RNA-sequencing datasets from secretory-phase endometrial biopsies from patients with severe endometriosis (GSE213216) and controls (GSE214411). (**A**) Proportion of immune cells relative to total sequenced cells. (**B**) Distribution of specific immune cell populations within the total cellular compartment.

**Figure 7:**
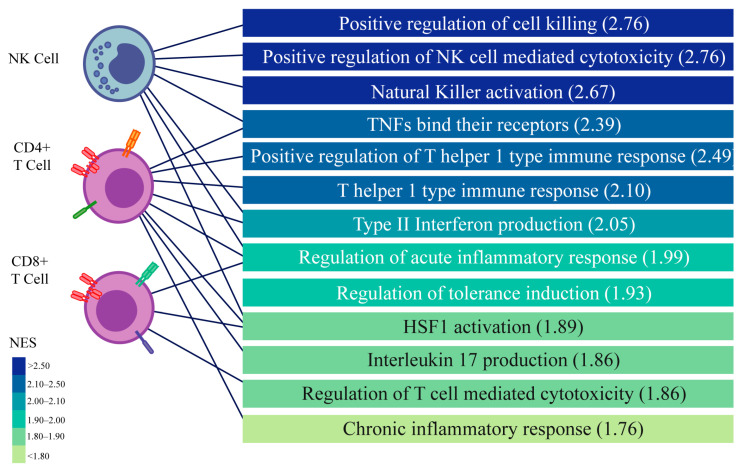
Endometrial T cells and NK cells from patients with severe endometriosis exhibit transcriptional shifts in biological processes. Single-cell RNA-sequencing data from secretory-phase eutopic endometrial biopsies from patients with severe endometriosis and healthy controls were used to identify T lymphocytes and NK cells in silico. Gene expression profiles of these cell populations were analyzed for functional enrichment using Gene Ontology Biological Process (GOBP), revealing significantly enriched pathways (FDR < 0.01).

Taking into account that endometriosis is considered an inflammatory disease, and that our results show an increase in inflammation on the eutopic endometrium that could affect implantation, we studied the role of the immune cells in the endometrial–embryo dialogue. In order to achieve this, we used transcriptomic data from single-cell RNAseq datasets from secretory-phase eutopic endometrial biopsies from patients with severe endometriosis (GSE213216), mild (GSE214411), and no-endometriosis controls (GSE214411). From these datasets, we identified cell types and found that the endometrium from patients with both mild and severe endometriosis had an increased proportion of immune cells, being NK and T cells the ones with higher increase (Figure 6). When we performed a GSEA on each of these cells, we observed a significant enrichment in pathways associated with inflammation, such as NK and Th1 activation. This finding goes along with our previous observations at the bulk endometrial level (Figure 7).

**Figure 8:**
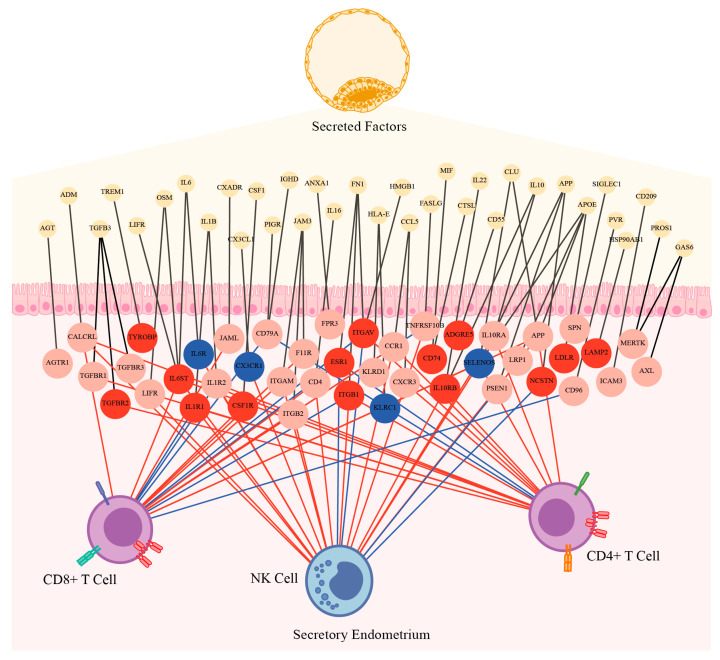
Differentially expressed genes in endometrial T cells and NK cells from patients with severe endometriosis intersect with embryo–endometrium interaction networks. Protein–protein interaction graph of membrane receptors expressed in endometrial biopsies during the secretory phase (GSE141549) up-regulated (red circles) and down-regulated (blue circles) genes in patients with severe endometriosis, with soluble factors expressed in day 5 embryos (GSE18290) and soluble factors significantly upregulated (red edges) or downregulated (blue edges) in T lymphocytes and NK cells identified via single-cell RNA-sequencing (GSE213216 and GSE214411) from secretory-phase endometrial tissue.

Since we found an increase in the proportion of immune cells expressing genes associated with a more inflammatory activation, we wondered if these immune cells could affect the dialogue between the endometrium and the blastocyst. To analyze this, we evaluated the secretome of NK cells, CD4^+^, and CD8^+^ T cells using the same methodology as for the blastocyst (Figure S4) and then analyzed the interactions with the endometrial receptors involved in immune pathways. As expected, we found that the studied immune cells produced soluble factors that could interact with the endometrial cell receptors. Furthermore, these interactions were significantly different on patients with endometriosis for 29 out of 44 endometrial receptors (65.9%, Figure 8), affecting the endometrial–embryo dialogue.

## 4. Discussion

Successful pregnancy requires a precise and timely interaction between a developing blastocyst and a receptive endometrium during the mid-secretory phase of the menstrual cycle [29]. Endometriosis has been associated with infertility, although the specific mechanisms involved are still not completely understood [5]. Furthermore, accumulating evidence indicates that endometriosis could create an altered and potentially dysfunctional microenvironment within the eutopic endometrium, which can hinder this crucial implantation process [30,31,32].

At the starting point of this manuscript there is a retrospective study which we performed on patients that underwent IVF with PGT-studied blastocysts in order to only evaluate the endometrial factor. We found that patients with endometriosis required significantly more attempts to achieve implantation which is in line with previous reports associating endometriosis with infertility [5,31]. However, once implantation was achieved, we found no significant difference in the pregnancy outcome between the two groups. Hence, the negative effect of endometriosis on fertility might involve the early stages of implantation. Unfortunately, human embryo implantation is commonly referred as a “black box” due to the limitations, both technical and ethical, that involve its study [33]. Therefore, in order to obtain insights of the processes involved in the early embryo–endometrial dialogue and how it is affected on patients with endometriosis, here we opted for a bioinformatic approach, integrating transcriptomic data from different sources.

Successful implantation requires a finely tuned molecular dialogue between the blastocyst and the receptive endometrium, which orchestrates a dynamic microenvironment involving tissue remodelling, angiogenesis, and immune modulation. This bidirectional communication is essential for establishing a pro-implantatory niche capable of supporting embryo invasion and maternal tolerance [5,34,35]. This is reflected upon the results of our interactome network as some of the most enriched pathways include angiogenesis, regulation of cell adhesion, regulation of the ECM, chemotaxis and regulation of NK, and T cells immunity. Interestingly, in recent years, it has been proposed that the decidual cells actively evaluate the quality of the blastocyst during implantation, allowing only the implantation of “high quality blastocysts” while limiting the “low quality” ones [16,36]. The interactome network presented here provides a molecular framework for this selective process, highlighting specific ligand–receptor interactions that may mediate endometrial sensing and response to embryonic signals.

When comparing the gene expression of endometrium from healthy patients vs. eutopic endometrium from patients with severe endometriosis, we found that most of the upregulated pathways were related to cell death, inflammation, and stress response. This last process, and particularly the UPR pathway, is interesting as it is directly involved in the decidualization program and in the induction of the sterile inflammation required for implantation [37,38,39,40]. As expected from these results, we found several genes that were significantly modulated in the endometrium of patients with endometriosis, further suggesting endometriotic patients have an altered embryo–endometrium dialogue, even when only considering the eutopic endometrium.

Considering the systemic inflammatory effects of endometriosis and the alteration we found associated with inflammation, we focused on the role of the immune cells in this dialogue [3]. We found that both, minimal/mild and severe endometriosis patients present an increased proportion of immune cells in the endometrium, indicating that, although the benefits of surgical treatment of minimal/mild endometriosis patients is disputed, the immune response in their endometria is altered and may affect their receptivity [41,42,43]. Importantly, these immune cells exhibited a transcriptomic profile enriched in inflammatory pathways, contrasting with the immune landscape of a healthy endometrium, which is typically associated with controlled inflammation, angiogenesis, and tissue remodelling during implantation [35,44]. The inflammatory activation of these cells may compromise endometrial receptivity by disrupting the tightly regulated immune tolerance required for successful implantation. In our dataset, CD4^+^ T cells showed increased expression of Th1-associated transcripts and IL-17, suggesting a pro-inflammatory Th1/Th17 profile. CD8^+^ T cells and NK cells exhibited upregulation of cytotoxicity-related genes, indicating enhanced effector activity. While these findings point to the immune-mediated disruption of the implantation environment, the precise identification of functional subtypes was beyond the resolution of our current analysis. Although previous studies have proposed roles for these subsets in implantation, further research is needed to define their specific contributions in the pathogenesis of endometriosis. In this sense, although all evidence aims to an immunological imbalance, the alterations of inflammatory and regulatory cells and mediators have contradictory reports [45,46]. A more granular and integrative characterization of immune subpopulations using scRNAseq would require larger sample sizes and dedicated analytical pipelines. Such studies would be highly valuable to elucidate the immunological mechanisms underlying endometriosis-associated infertility.

Soluble factors secreted by identified immune populations interacted with endometrial receptors involved in the embryo–endometrium dialogue, and many of these interactions were significantly altered in endometriosis patients. This suggests that immune-mediated interference may be a key contributor to the impaired implantation observed in endometriosis-associated infertility [5,6,31].

Despite the growing evidence, the underlying molecular mechanisms involved in endometriosis-associated infertility are still unclear, especially in the very early stages of implantation. Our integrative bioinformatic approach, although limited by its dependence on publicly available transcriptomic datasets, allowed us to identify key molecular pathways and cell populations potentially involved in the impaired embryo–endometrium dialogue. These findings require experimental validation in functional models to confirm the biological relevance of the identified interactions and their impact on implantation outcomes.

Our study identified immune-related and tissue remodelling pathways disrupted in the eutopic endometrium of endometriosis patients, highlighting ligand–receptor interactions that may impair early implantation. These findings suggest potential targets for clinical intervention and warrant experimental validation in future studies.

## Data Availability

Transcriptomic data are publicly available at GEO (https://www.ncbi.nlm.nih.gov/geo/) under accessions: GSE141549, GSE18290, GSE213216 and GSE214411.

## Supplementary Materials

The following supporting information can be downloaded at: https://www.mdpi.com/article/10.3390/biomedicines13112588/s1. The following figures are available as supplementary material: Figure S1. Global interaction network between endometrial receptors and embryo-derived soluble factors highlights extensive molecular crosstalk. Figure S2. Some of the soluble factors expressed by the blastocyst are capable of regulating endometrial functions independently of direct receptor-mediated interactions. Figure S3. Differentially expressed genes in the eutopic endometrium from patients with severe endometriosis intersect with embryo–endometrium interaction networks. Figure S4. Immune cells residing in the eutopic endometrium of patients with endometriosis show an altered gene expression pattern. Table S1. Curated ligand-receptor interactions detected by STRING and the main bio-logical process associated to each gene. Table S2. Details of the significantly enriched pathways detected in the interactome between blastocyst and endometrial tissue.
