# Supplementary material for: Exploring the Early Endometrial–Blastocyst Interactome in Endometriosis: An Integrative Study
Source: Biomedicines. 2025 Oct 23;13(11):2588. doi: 10.3390/biomedicines13112588 (PMC12649848; doi:10.3390/biomedicines13112588)
Supplement: Supplementary file 1 [file biomedicines-13-02588-s001.zip › supplementary material - figures S1-S4.pdf]

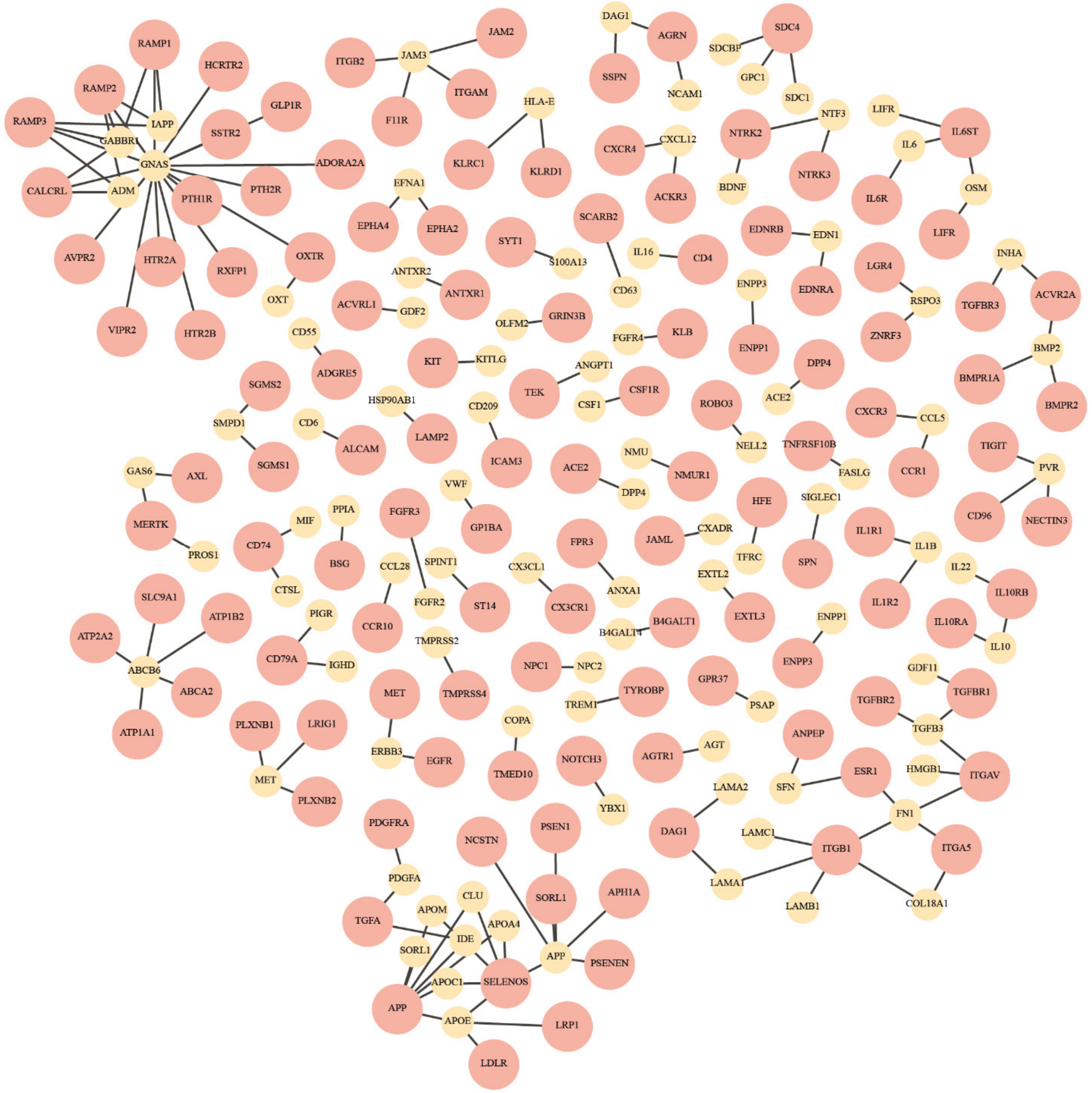

Figure S1. Global interaction network between endometrial receptors and embryo-derived soluble factors highlights extensive molecular crosstalk. Protein–protein interaction network constructed from membrane receptor genes expressed at the mRNA level in endometrial biopsies (GSE141549) and soluble ligand genes expressed in day 5 human embryos (GSE18290).

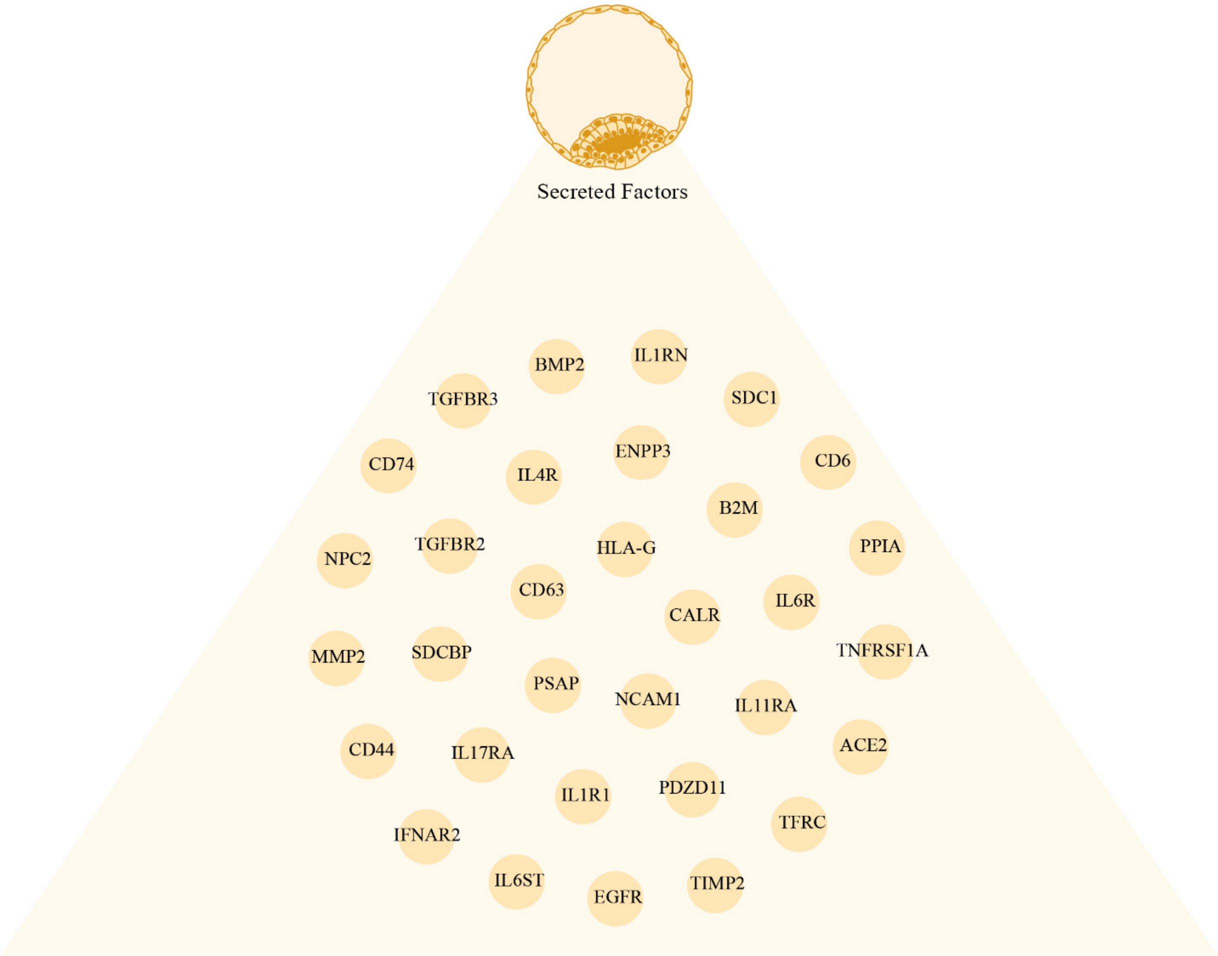

Figure S2. Some of the soluble factors expressed by the blastocyst are capable of regulating endometrial functions independently of direct receptor-mediated interactions. Transcriptomic data from day 5 human embryos (GSE18290) were used to identify soluble ligands expressed at the mRNA level. These factors may exert regulatory effects on the endometrium through indirect or receptor-independent mechanisms.

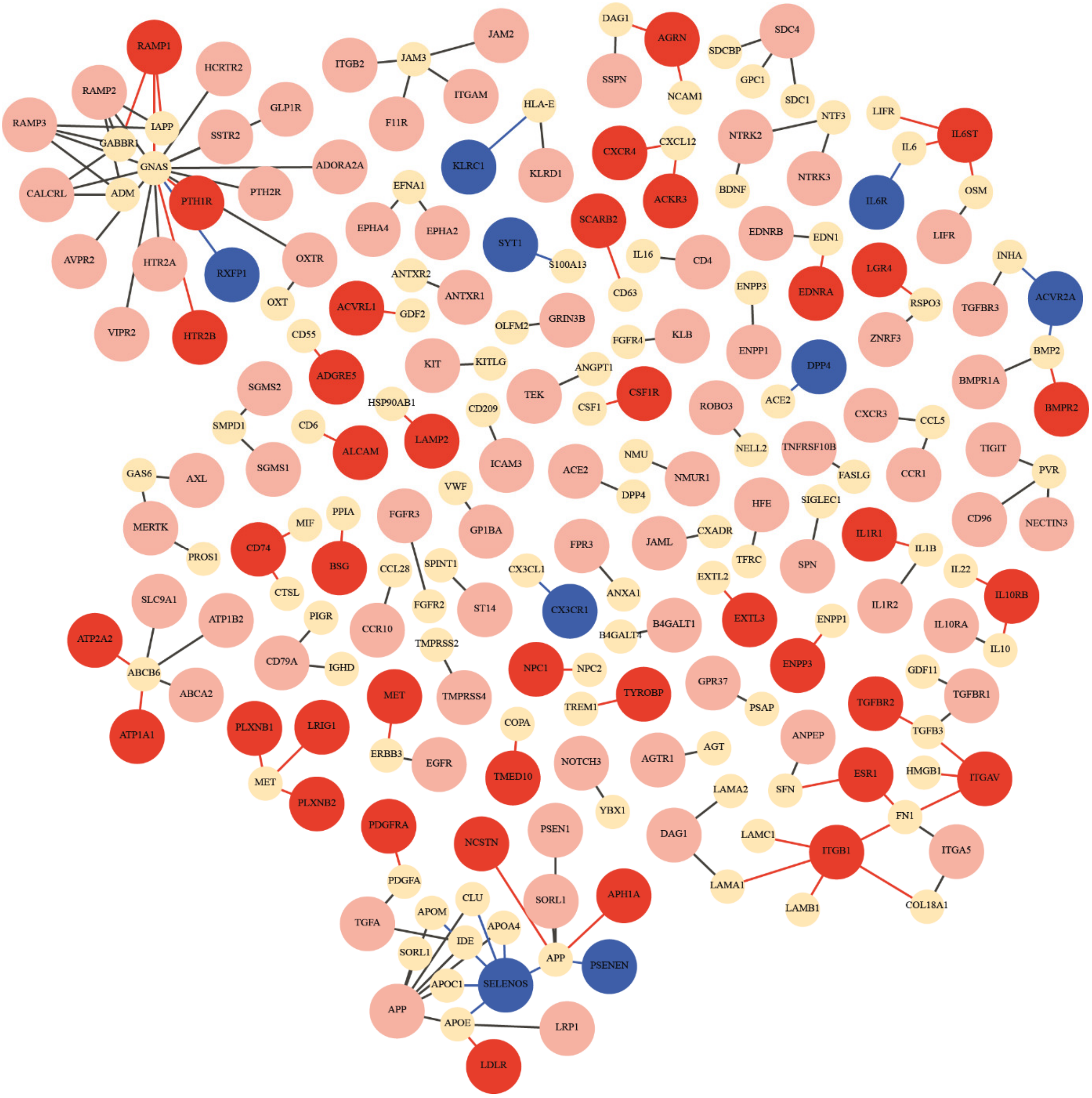

Figure S3. Differentially expressed genes in the eutopic endometrium of patients with severe endometriosis intersect with embryo–endometrium interaction networks. Protein–protein interaction network constructed from membrane receptor genes expressed at the mRNA level in endometrial biopsies (GSE141549) and soluble ligand genes expressed in day 5 human embryos (GSE18290). Genes significantly upregulated (red edges) or downregulated (blue edges) in patients with severe endometriosis compared to healthy controls (|fold change| > 2; FDR < 0.01) were mapped onto the network

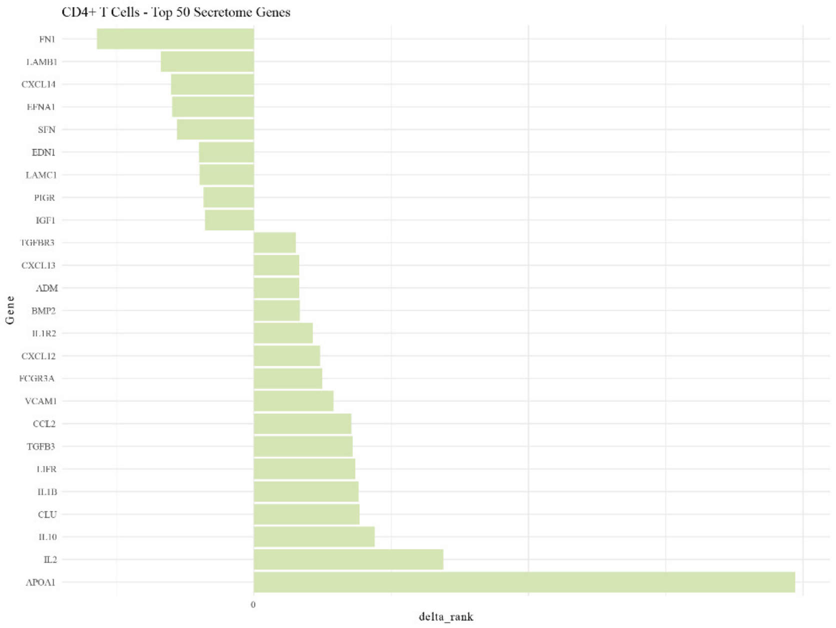
CD4+ T Cells - Top 50 Secretome Genes
FN1
LAMB1
CXCL14
EFNA1
SFN
EDN1
LAMC1
PIGR
IGF1
TGFBR3
CXCL13
ADM
BMP2
IL1R2
CXCL12
FCGR3A
VCAM1
CCL2
TGFB3
LIFR
IL1B
CLU
IL10
IL2
APOA1
Gene
0
delta_rank

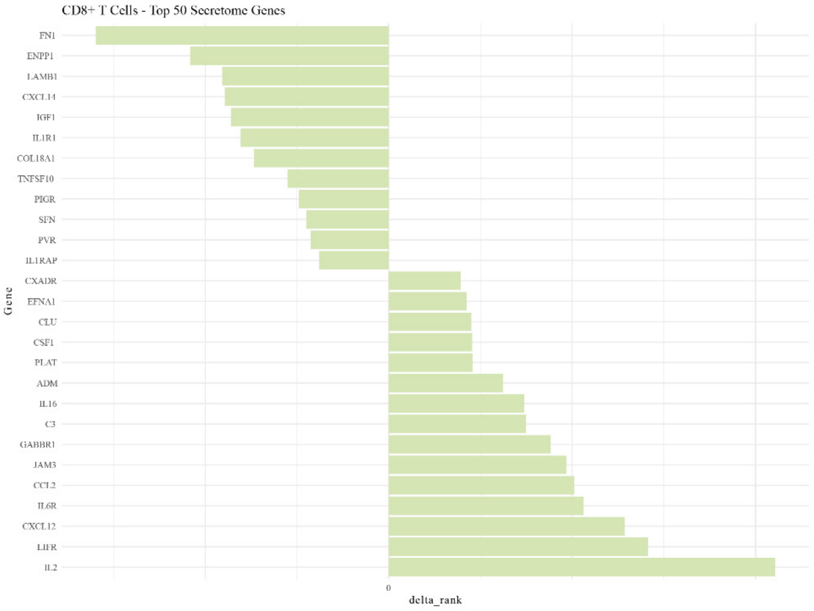
CD8+ T Cells - Top 50 Secretome Genes
FN1
ENPP1
LAMB1
CXCL14
IGF1
IL1R1
COL18A1
TNFSF10
PIGR
SFN
PVR
IL1RAP
CXADR
EFNA1
CLU
CSF1
PLAT
ADM
IL16
C3
GABBR1
JAM3
CCL2
IL6R
CXCL12
LIFR
IL2
Gene
0
delta_rank

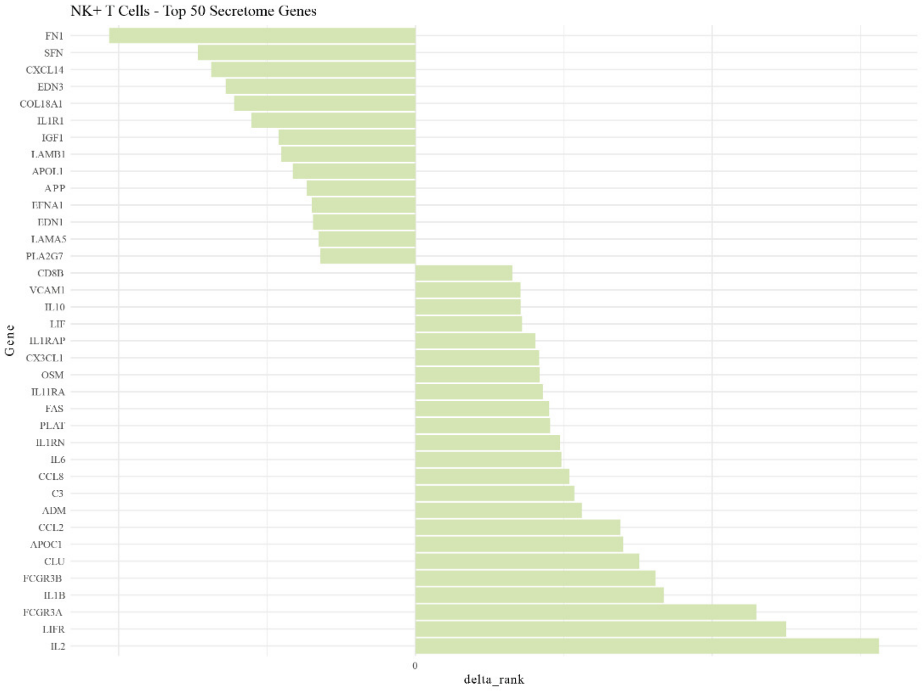

Figure S4. Immune cells resident in the eutopic endometrium of patients with endometriosis show an altered gene expression pattern. Differentially expressed genes (delta_rank > |1500|) identified in CD4+ T cells (A), CD8+ T cells (B), and NK cells (C) from secretory-phase endometrial tissue were selected based on their annotation as secreted factors. These genes participate in the protein–protein interaction network described in Figure 8, connecting immune-derived signals with endometrial receptors involved in embryo communication.
