# Supplementary material for: Exploring the Early Endometrial–Blastocyst Interactome in Endometriosis: An Integrative Study
Source: Biomedicines. 2025 Oct 23;13(11):2588. doi: 10.3390/biomedicines13112588 (PMC12649848; doi:10.3390/biomedicines13112588)
Supplement: Supplementary file 1 [file biomedicines-13-02588-s001.zip › Supplementary material - Table S1.pdf]

Table S1.

Ligand	Lingand associated process	Receptor	Receptor associated process	Regulation on Endometriosis
ABCB6	Others	ATP1B2	Adhesion	NonReg
ABCB6	Others	ATP2A2	Adhesion	UpReg
ABCB6	Others	SLC9A1	Adhesion	NonReg
ABCB6	Others	ATP1A1	Others	UpReg
ABCB6	Others	ABCA2	Others	NonReg
ACE2	Inflammation	DPP4	Adhesion	DownReg
ADM	Inflammation	CALCRL	Inflammation	NonReg
ADM	Inflammation	RAMP2	Adhesion	NonReg
ADM	Inflammation	RAMP3	Others	NonReg
AGT	Inflammation	AGTR1	Inflammation	NonReg
ANGPT1	Adhesion	TEK	Adhesion and Inflammation	NonReg
ANTXR2	Others	ANTXR1	Adhesion	NonReg
ANXA1	Adhesion and Inflammation	FPR3	Inflammation	NonReg
APOA4	Adhesion	APP	Adhesion and Inflammation	NonReg
APOA4	Adhesion	SELENOS	Inflammation	DownReg
APOC1	Others	APP	Adhesion and Inflammation	NonReg
APOC1	Others	SELENOS	Inflammation	DownReg
APOE	Adhesion and Inflammation	APP	Adhesion and Inflammation	NonReg
APOE	Adhesion and Inflammation	LDLR	Inflammation	UpReg
APOE	Adhesion and Inflammation	LRP1	Inflammation	NonReg
APOE	Adhesion and Inflammation	SELENOS	Inflammation	DownReg
APOM	Others	APP	Adhesion and Inflammation	NonReg
APOM	Others	SELENOS	Inflammation	DownReg
APP	Adhesion and Inflammation	NCSTN	Adhesion and Inflammation	UpReg
APP	Adhesion and Inflammation	PSEN1	Adhesion and Inflammation	NonReg
APP	Adhesion and Inflammation	SELENOS	Inflammation	DownReg
APP	Adhesion and Inflammation	PSENEN	Others	DownReg
APP	Adhesion and Inflammation	SORL1	Others	NonReg
APP	Adhesion and Inflammation	APH1A	Others	UpReg
B4GALT4	Others	B4GALT1	Adhesion and Inflammation	NonReg
BDNF	Adhesion	NTRK2	Others	NonReg
BMP2	Adhesion and Inflammation	BMPR1A	Others	NonReg
BMP2	Adhesion and Inflammation	BMPR2	Others	UpReg
BMP2	Adhesion and Inflammation	ACVR2A	Others	DownReg
CCL28	Adhesion	CCR10	Others	NonReg
CCL5	Adhesion and Inflammation	CCR1	Adhesion and Inflammation	NonReg
CCL5	Adhesion and Inflammation	CXCR3	Adhesion and Inflammation	NonReg
CD209	Adhesion and Inflammation	ICAM3	Adhesion and Inflammation	NonReg
CD55	Adhesion and Inflammation	ADGRE5	Adhesion and Inflammation	UpReg
CD6	Adhesion and Inflammation	ALCAM	Adhesion	UpReg
CD63	Adhesion and Inflammation	SCARB2	Others	UpReg
CLU	Inflammation	APP	Adhesion and Inflammation	NonReg

CLU	Inflammation	SELENOS	Inflammation	DownReg
COL18A1	Adhesion	ITGB1	Adhesion and Inflammation	UpReg
COL18A1	Adhesion	ITGA5	Adhesion	NonReg
COPA	Others	TMED10	Others	UpReg
CSF1	Adhesion and Inflammation	CSF1R	Adhesion and Inflammation	UpReg
CTSL	Adhesion and Inflammation	CD74	Adhesion and Inflammation	UpReg
CX3CL1	Adhesion and Inflammation	CX3CR1	Adhesion and Inflammation	DownReg
CXADR	Adhesion and Inflammation	JAML	Adhesion and Inflammation	NonReg
CXCL12	Adhesion	CXCR4	Adhesion and Inflammation	UpReg
CXCL12	Adhesion	ACKR3	Adhesion	UpReg
DAG1	Adhesion	SSPN	Adhesion	NonReg
DAG1	Adhesion	AGRN	Adhesion	UpReg
DPP4	Adhesion	ACE2	Inflammation	NonReg
EDN1	Others	EDNRB	Inflammation	NonReg
EDN1	Others	EDNRA	Others	UpReg
EFNA1	Adhesion	EPHA2	Adhesion and Inflammation	NonReg
EFNA1	Adhesion	EPHA4	Adhesion	NonReg
ENPP1	Others	ENPP3	Inflammation	UpReg
ENPP3	Inflammation	ENPP1	Others	NonReg
ERBB3	Adhesion	EGFR	Adhesion	NonReg
ERBB3	Adhesion	MET	Others	UpReg
EXTL2	Others	EXTL3	Others	UpReg
FASLG	Inflammation	TNFRSF10B	Inflammation	NonReg
FGFR2	Others	FGFR3	Others	NonReg
FGFR4	Others	KLB	Others	NonReg
FN1	Adhesion and Inflammation	ITGAV	Adhesion and Inflammation	UpReg
FN1	Adhesion and Inflammation	ITGB1	Adhesion and Inflammation	UpReg
FN1	Adhesion and Inflammation	ESR1	Inflammation	UpReg
FN1	Adhesion and Inflammation	ITGA5	Adhesion	NonReg
GABBR1	Others	CALCRL	Inflammation	NonReg
GABBR1	Others	RAMP2	Adhesion	NonReg
GABBR1	Others	RAMP1	Others	UpReg
GABBR1	Others	RAMP3	Others	NonReg
GAS6	Adhesion	AXL	Adhesion and Inflammation	NonReg
GAS6	Adhesion	MERTK	Adhesion	NonReg
GDF11	Others	TGFBR1	Adhesion and Inflammation	NonReg
GDF2	Others	ACVRL1	Adhesion	UpReg
GNAS	Adhesion	ADORA2A	Adhesion and Inflammation	NonReg
GNAS	Adhesion	CALCRL	Inflammation	NonReg
GNAS	Adhesion	RAMP2	Adhesion	NonReg
GNAS	Adhesion	RXFP1	Adhesion	DownReg
GNAS	Adhesion	HCRTR2	Others	NonReg
GNAS	Adhesion	HTR2A	Others	NonReg
GNAS	Adhesion	HTR2B	Others	UpReg
GNAS	Adhesion	OXTR	Others	NonReg

GNAS	Adhesion	PTH1R	Others	UpReg
GNAS	Adhesion	PTH2R	Others	NonReg
GNAS	Adhesion	RAMP1	Others	UpReg
GNAS	Adhesion	RAMP3	Others	NonReg
GNAS	Adhesion	SSTR2	Others	NonReg
GNAS	Adhesion	VIPR2	Others	NonReg
GNAS	Adhesion	AVPR2	Others	NonReg
GNAS	Adhesion	GLP1R	Others	NonReg
GPC1	Others	SDC4	Adhesion	NonReg
HLA-E	Adhesion and Inflammation	KLRC1	Inflammation	DownReg
HLA-E	Adhesion and Inflammation	KLRD1	Inflammation	NonReg
HMGB1	Adhesion and Inflammation	ITGAV	Adhesion and Inflammation	UpReg
HSP90AB1	Inflammation	LAMP2	Inflammation	UpReg
IAPP	Others	RAMP2	Adhesion	NonReg
IAPP	Others	RAMP1	Others	UpReg
IAPP	Others	RAMP3	Others	NonReg
IDE	Others	APP	Adhesion and Inflammation	NonReg
IDE	Others	TGFA	Others	NonReg
IGHD	Inflammation	CD79A	Inflammation	NonReg
IL10	Inflammation	IL10RA	Inflammation	NonReg
IL10	Inflammation	IL10RB	Inflammation	UpReg
IL16	Inflammation	CD4	Adhesion and Inflammation	NonReg
IL1B	Adhesion and Inflammation	IL1R1	Inflammation	UpReg
IL1B	Adhesion and Inflammation	IL1R2	Inflammation	NonReg
IL22	Inflammation	IL10RB	Inflammation	UpReg
IL6	Adhesion and Inflammation	IL6R	Adhesion and Inflammation	DownReg
IL6	Adhesion and Inflammation	IL6ST	Adhesion and Inflammation	UpReg
INHA	Adhesion	TGFBR3	Adhesion and Inflammation	NonReg
INHA	Adhesion	ACVR2A	Others	DownReg
JAM3	Adhesion and Inflammation	F11R	Adhesion and Inflammation	NonReg
JAM3	Adhesion and Inflammation	ITGAM	Adhesion and Inflammation	NonReg
JAM3	Adhesion and Inflammation	ITGB2	Adhesion and Inflammation	NonReg
JAM3	Adhesion and Inflammation	JAM2	Adhesion	NonReg
KITLG	Adhesion	KIT	Adhesion and Inflammation	NonReg
LAMA1	Adhesion	ITGB1	Adhesion and Inflammation	UpReg
LAMA1	Adhesion	DAG1	Adhesion	NonReg
LAMA2	Adhesion	DAG1	Adhesion	NonReg
LAMB1	Adhesion	ITGB1	Adhesion and Inflammation	UpReg
LAMC1	Adhesion	ITGB1	Adhesion and Inflammation	UpReg
LIFR	Inflammation	IL6ST	Adhesion and Inflammation	UpReg
MET	Others	PLXNB1	Adhesion	UpReg
MET	Others	PLXNB2	Adhesion	UpReg
MET	Others	LRIG1	Others	UpReg
MIF	Inflammation	CD74	Adhesion and Inflammation	UpReg
NCAM1	Adhesion and Inflammation	AGRN	Adhesion	UpReg

NELL2	Others	ROBO3	Adhesion	NonReg
NMU	Others	NMUR1	Others	NonReg
NPC2	Inflammation	NPC1	Others	UpReg
NTF3	Others	NTRK2	Others	NonReg
NTF3	Others	NTRK3	Others	NonReg
OLFM2	Others	GRIN3B	Others	NonReg
OSM	Inflammation	IL6ST	Adhesion and Inflammation	UpReg
OSM	Inflammation	LIFR	Inflammation	NonReg
OXT	Others	OXTR	Others	NonReg
PDGFA	Adhesion	PDGFRA	Adhesion	UpReg
PDGFA	Adhesion	TGFA	Others	NonReg
PIGR	Inflammation	CD79A	Inflammation	NonReg
PPIA	Adhesion and Inflammation	BSG	Adhesion	UpReg
PROS1	Inflammation	MERTK	Adhesion	NonReg
PSAP	Inflammation	GPR37	Others	NonReg
PVR	Adhesion and Inflammation	CD96	Adhesion and Inflammation	NonReg
PVR	Adhesion and Inflammation	TIGIT	Adhesion	NonReg
PVR	Adhesion and Inflammation	NECTIN3	Adhesion	NonReg
RSPO3	Others	ZNRF3	Others	NonReg
RSPO3	Others	LGR4	Others	UpReg
S100A13	Others	SYT1	Others	DownReg
SDC1	Adhesion and Inflammation	SDC4	Adhesion	NonReg
SDCBP	Adhesion and Inflammation	SDC4	Adhesion	NonReg
SFN	Others	ANPEP	Inflammation	NonReg
SFN	Others	ESR1	Inflammation	UpReg
SIGLEC1	Adhesion and Inflammation	SPN	Adhesion and Inflammation	NonReg
SMPD1	Others	SGMS1	Others	NonReg
SMPD1	Others	SGMS2	Others	NonReg
SORL1	Others	APP	Adhesion and Inflammation	NonReg
SPINT1	Adhesion	ST14	Others	NonReg
TFRC	Adhesion and Inflammation	HFE	Adhesion	NonReg
TGFB3	Adhesion and Inflammation	TGFBR1	Adhesion and Inflammation	NonReg
TGFB3	Adhesion and Inflammation	TGFBR2	Adhesion and Inflammation	UpReg
TGFB3	Adhesion and Inflammation	ITGAV	Adhesion and Inflammation	UpReg
TMPRSS2	Others	TMPRSS4	Others	NonReg
TREM1	Inflammation	TYROBP	Inflammation	UpReg
VWF	Adhesion	GP1BA	Adhesion	NonReg
YBX1	Others	NOTCH3	Others	NonReg

Table S1: Curated ligand-receptor interactions detected by STRING and the main biological process associated to each gene. Regulation of the receptor in endometrial samples from endometriotic patients is annotated as UpReg (significantly up-regulated in endometriosis), NonReg (no significant modulation) and DownReg (significantly down-regulated in endometriosis).
