# Supplementary material for: Exploring the Early Endometrial–Blastocyst Interactome in Endometriosis: An Integrative Study
Source: Biomedicines. 2025 Oct 23;13(11):2588. doi: 10.3390/biomedicines13112588 (PMC12649848; doi:10.3390/biomedicines13112588)
Supplement: Supplementary file 1 [file biomedicines-13-02588-s001.zip › Supplementary material - Table S2.pdf]

Table S2.

Data base	Term description	Strength	Signal	FDR	Matching proteins in the network
Reactome	Integrin cell surface interactions	1.17	2.09	1.06e-10	ICAM3, ITGAV, VWF, ITGA5, JAM3, BSG, FN1, COL18A1, F11R, AGRN, ITGB1, ITGB2, JAM2, ITGAM
Reactome	Laminin interactions	1.29	1.25	9.50e-06	LAMB1, LAMC1, ITGAV, COL18A1, LAMA1, ITGB1, LAMA2
Reactome	Degradation of the extracellular matrix	0.82	0.89	6.22e-05	LAMB1, LAMC1, NCSTN, PSEN1, BSG, CTSL, FN1, COL18A1
GOBP	Regulation of cell adhesion	0.81	2.51	8.44e-29	CX3CL1, CD74, CD4, LAMB1, LAMC1, ITGAV, IL1B, SLC9A1, ERBB3, EPHA4, ITGA5, JAM3, CD209, CD6, CSF1, GP1BA, HMGB1, FN1, EPHA2, PLXNB1, TGFBR2, PLXNB2, DPP4, CCL28, CD55, F11R, EFNA1, IL6R, SDC4, CXCR3, HLA-E, ANXA1, BMP2, TEK, IL6ST, ACVRL1, LAMA1, TFRC, CXCL12, ITGB2, IL6, CXCR4, LAMA2, IL10, HFE, TIGIT, ANGPT1, DAG1, CCL5, ADORA2A, KITLG, SPN, HFE, TIGIT, ANGPT1, DAG1, CCL5, ADORA2A, KITLG, B2M, SPN
GOBP	Regulation of extracellular matrix organization	1.29	2.25	7.89e-11	LAMB1, LRP1, LAMC1, FGFR4, ANTXR1, DPP4, AGT, BMP2, LAMA1, IL6, LAMA2, DAG1
GOBP	Regulation of actin cytoskeleton reorganization	1.07	0.83	0.00041	PDGFRA, CSF1R, NTRK3, F11R, TEK, NTF3
KEGG	Focal adhesion	0.81	1.2	3.70e-07	LAMB1, PDGFRA, LAMC1, ITGAV, VWF, EGFR, ITGA5, MET, PDGFA, FN1, LAMA1, ITGB1, LAMA2
KEGG	Adherens junction	0.86	0.67	0.0014	EGFR, MET, TGFBR2, NECTIN3, TGFBR1
GOBP	Cell chemotaxis	1.11	3.28	3.08e-22	CX3CL1, TREM1, PDGFRA, IL1B, ACKR3, CXADR, KIT, CCR1, IL16, GAS6, CCR10, BSG, HMGB1, JAML, CX3CR1, EPHA2, CCL28, IL6R, CXCR3, ANXA1, EDNRB, CXCL12, ITGB2, IL6, CXCR4, AGTR1, IL10, PPIA, CCL5
GOBP	Angiogenesis	0.94	2.52	5.23e-19	CX3CL1, RAMP2, RAMP1, PDGFRA, ITGAV, NOTCH3, ACKR3, ITGA5, JAM3, ANPEP, EDNRA, BSG, PDGFA, FN1, RSPO3, EPHA2, TGFBR2, COL18A1, EFNA1, CXCR3, EDN1, B4GALT1, TEK, ACVRL1, CALCRL, ITGB1, TGFA, FGFR2, ANGPT1, DAG1, TGFBR1, GDF2, GDF2
GOBP	Regulation of T cell mediated immunity	1.07	1.53	7.27e-08	IL1B, KLRD1, HMGB1, CD55, HLA-E, IL6, IL1R1, PVR, HFE, KLRC1
GOBP	Negative regulation of natural killer cell mediated immunity	1.32	0.96	0.00019	CD96, KLRD1, HLA-E, KLRC1
KEGG	Antigen processing and presentation	1.2	1.92	1.67e-09	CD74, CD4, KLRD1, CTSL, HSP90AB1, HLA-E, KLRC1
Reactome	Signaling by Interleukins	0.82	2.02	2.80e-16	CD4, MIF, OSM, IL10RA, IL1B, APP, CSF1R, IL10RB, CCR1, IL16, CSF1, IL1R2, HMGB1, FN1, FASLG, IL6R, ANXA1, SDC1, IL6ST, ITGB1, ITGB2, IL6, IL1R1, LIFR, IL10, PPIA, IL22, CCL5, ITGAM, IL11RA, CCL5, ITGAM

Table S2: Details of the significantly enriched pathways detected in the interactome between blastocyst and endometrial tissue. The genes shown for each pathway, correspond to genes that belong to the pathway and are present in the network.
